# Cognitive Remediation Therapy for Brain Tumor Survivors with Cognitive Deficits

**DOI:** 10.7759/cureus.350

**Published:** 2015-10-13

**Authors:** Amanda Sacks-Zimmerman, Devika Duggal, Taylor Liberta

**Affiliations:** 1 Neurological Surgery, Weill Cornell Medicine; 2 Neurological Surgery, Weill Cornell Medical College

**Keywords:** low-grade glioma, continuity of care, cognitive remediation, computerized cognitive remediation, cognitive deficits, working memory, brain tumor survivorship

## Abstract

Cognitive deficits have been widely observed in patients with primary brain tumors consequent to diagnosis and treatment. Given the early onset and the relatively long survival rate of patients, it seems pertinent to study and refine the techniques used to treat these deficits. The purpose of this article is to discuss cognitive deficits that follow neurosurgical treatment for low-grade gliomas as well as to outline a neuropsychological intervention to treat these deficits, specifically working memory and attention. Cognitive remediation therapy is a neuropsychological intervention that aims to enhance attention, working memory, and executive functioning, thereby diminishing the impact of these deficits on daily functioning. Computerized cognitive remediation training programs facilitate access to treatment through providing online participation. The authors include preliminary results of three participants who have completed the computerized training program as part of an ongoing study that is investigating the efficacy of this program in patients who have undergone treatment for low-grade gliomas. The results so far suggest some improvement in working memory and attention from baseline scores. It is the hope of the present authors to highlight the importance of this treatment in the continuity of care of brain tumor survivors.

## Introduction and background

### Cognitive deficits

Cognitive function is now recognized as a critical outcome measure in patients with primary brain tumors, such as low-grade gliomas [1]. These patients can typically show impaired functioning in cognitive domains like attention, memory, executive function, and graphomotor speed [2].

Cognitive deficits in patients with brain tumors are primarily caused by the tumor location, tumor-related epilepsy, and tumor treatment [3]. Additionally, psychological distress, including anxiety and depression, can exacerbate cognitive deficits [4]. While the data available on cognitive deficits of patients prior to treatment is limited, there are studies that demonstrate cognitive impairments at the time of diagnosis. For instance, Tucha and his colleagues [5] found 90% of pre-treatment patients with frontal or temporal lobe tumors displayed impairments in at least one area of cognition. Other studies suggest that while the tumors themselves have an adverse effect on cognitive functioning, treatment modalities like radiotherapy [6] and adjuvant chemotherapy [7] exacerbate existing deficits, resulting in more long-term disability. It is most likely, however, that combinations of the above, along with other factors, such as tumor regrowth, leptomeningeal metastasis, or metabolic disturbances, contribute to cognitive impairment [3].

While tumor-induced cognitive deficits are linked to tumor size and location [8], post-treatment impairments are usually diffuse, independent of tumor location and can manifest six months to years after completion of treatment [9]. Neurosurgical interventions may cause neurological deficits due to damage to the surrounding tissue. Radiation-induced cognitive impairment has been reported to occur in 50-90% of tumor survivors [9-10] and is marked by decreased verbal and spatial memory, attention, and problem-solving abilities [11]. The mechanism of radiation-induced neurotoxicity has been attributed to demyelination and microvascular injury, leading to necrosis [12], inflammation, and impaired hippocampal neurogenesis [13]. The relative density of white matter tracts that may be affected by radiation therapy [14] are in the frontal and subcortical regions and contribute to deficits, including processing speed, attention, learning, memory, and executive functions [15]. Adjuvant chemotherapy has also been associated with impaired neurocognitive functioning. However, it is likely that the effects of both treatments are synergistic and are associated with long-term neurotoxicity [16].

Cognitive dysfunction is, therefore, a long-term issue for survivors and continuity of care is essential for their quality of life. Cognitive impairment is a major cause of disability for individuals with brain tumors and is consistently identified by patients and caregivers as the greatest source of hindrance to post-treatment quality of life [17]. These aforementioned deficits pose long-term concerns regarding survivors’ reintegration into active living. Cancer survivorship, living beyond cancer, refers to post-treatment and long-term survivorship. Therefore, cognitive remediation treatment is essential for survivorship, which includes facilitating reintegration into daily life. Treating these deficits, through cognitive remediation therapy (CRT), is crucial, and the development and research on effective treatment strategies deserve greater attention.

### Cognitive remediation therapy (CRT)

CRT, based on the principles of neural plasticity, is designed to improve neurocognitive abilities, such as attention, working memory, cognitive control, and executive function, leading to improved social and occupational functioning. In CRT, cognitive exercises are used to improve functioning in the affected areas. These exercises usually involve repetitive “drill and practice” computerized or pen-and-paper training in attention, memory, language, reasoning, and problem-solving. These exercises collectively comprise CRT and aim to restore impaired function and/or compensate for the area of deficit through strategy training and repeated skills practice [18].

Cognitive remediation typically relies on two types of exercises: retraining and compensation [19]. While retraining consists of regular, repeated practice of tasks that can strengthen impaired neurocognitive functions, compensation focuses on learning new strategies and alternative means to achieve goals, such as the use of planners, checklists, and memory notebooks for daily planning. These two forms of training work in tandem (restorative processes helping to develop compensatory strategies and vice versa) with the goal of increasing functionality [20]. CRT can be delivered through computerized programs or can be used by a trained clinician as a more individualized approach.

Computerized CRT is gaining popularity as it allows for greater access to therapy within the home environment. This form of treatment targets the two most prevalent areas of cognitive dysfunction found in post-neurosurgical patients: attention and working memory. Another advantage of these programs is that they provide immediate feedback and automatically adjust the task difficulty level based on performance. An important aspect of remediation addressed by computerized cognitive remediation programs is working memory. Working memory (WM) is a crucial aspect of cognitive remediation as it is part of the central executive system and underlies other cognitive abilities like memory. WM abilities are essential in several goal-oriented processes, such as the manipulation of incoming information and maintaining goal-directed behaviors in the face of interfering stimuli.

## Review

### Effectiveness of CRT

CRT has been effective in treating other patient populations, such as traumatic brain injury [21-22] and stroke patients [23], as well as other disorders where cognitive impairment is implicit, such as attention deficit hyperactivity disorder (ADHD) [24] and schizophrenia [25]. Other studies have also found CRT to be effective in remediating attention processes with pediatric cancer survivors [19, 26]. Researchers have indicated its efficacy in low-grade glioma (LGG) patients as well [27-28]. Using a multifaceted cognitive rehabilitation program (CRP), Gehring and colleagues [27] observed a significant increase in self-reported cognitive functioning at the immediate post-intervention assessment but not at the six-month follow-up. Conversely, significant increases were observed in the neuropsychological assessment of attention and verbal memory at the six-month follow-up as well as a reported decrease in mental fatigue [27]. Zucchella and colleagues [28] combined and administered individual sessions of therapist-guided cognitive training and computerized exercises over four weeks. At the post-intervention assessment, the investigators observed a significant improvement in cognitive functioning, particularly in visual attention and verbal memory [28].

Unlike stroke and brain injury, few studies have been conducted on the efficacy of cognitive rehabilitation on brain tumor survivors. While studies on CRT have shown measurable improvements in cognitive performance, the findings need to be observed across neurological disorders, including individuals with LGG post-surgery.

### Cogmed Working Memory Training® Program

The Cogmed Working Memory Training® is an evidence-based intervention program that aims at improving working memory. It consists of 25 training sessions that can be completed online, each session lasting 30-45 minutes and consisting of systematic exercises that target various aspects of working memory.

The present authors are currently investigating the efficacy of Cogmed® on patients experiencing cognitive impairments post-treatment for LGG. These tumors account for approximately 40% of all gliomas in adults and have a relatively long median survival rate of 10 years or more [29]. These tumors are usually diagnosed in adulthood, including working-age adults, and therefore, the resulting deficits negatively impact vocational and academic functioning. Cognitive deficits post-multimodal treatment in low-grade gliomas tend to be consistent with frontal-subcortical dysfunction; specifically, sustained attention, multi-tasking, organizing and sequencing tasks, processing speed, nonverbal recall, organizing information in memory, and visual-motor coordination and speed [30]. The present study is following 20 participants, all at least four months post-multimodal treatment for LGGs. Neuropsychological evaluations are conducted to establish baseline scores after which the participant is introduced to the Cogmed® five-week training program. The neuropsychological test battery consists of tests measuring performance on attention, working memory, memory, mood, and subjective functioning. On completing the Cogmed® training, the participants are tested with the aforementioned battery at two subsequent time points: within two weeks of completion in order to measure any changes in functioning post-intervention, and after three months to establish the maintenance of gains provided by the training.

The results of three participants who have completed the study are presented below, along with their demographic data, illness, and treatment information shown in Table 1. Signed informed consent was obtained for their participation in this study. Within each bar cluster is individual scores of each participant at three time points. For the purpose of the present paper, a subset of the test battery will be used to indicate any change in performance in the following domains:

1. Attention: Digit Span Forward (WAIS-IV), Rey’s Auditory Verbal Learning Test (RAVLT) Trial 1.

2. Working memory: Digit Span Backward (WAIS-IV), Brief Test of Attention-Letters, Letter-Number Sequencing (WAIS-IV).

3. Memory: RAVLT Short Delay Free Recall (SDFR) and Long Delay Free Recall (LDFR).

4. Mood: Beck Depression Inventory (BDI) and Beck Anxiety Inventory (BAI).

**Table 1:** Demographic, illness and treatment data for three participants at the time of enrollment

Participants	Sex	Age	Tumor Type	Treatment
1	M	63	Pilocytic astrocytoma	Partial resection
2	F	27	Pilocytic astrocytoma	Partial resection
3	M	53	Oligoastrocytoma	Gross total resection

**Figure 1:**
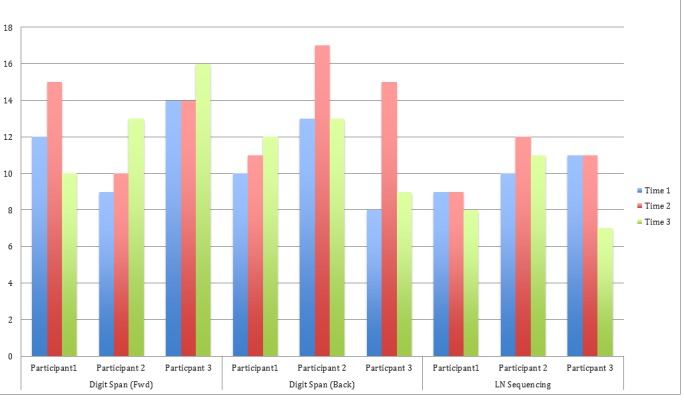
WAIS-IV Standard Scores for Three Participants Across Three Time Points

The results obtained so far suggest that Cogmed® may help improve scores in the aforementioned domains. In Figure 1, for digit span forward, participant 1 showed improved scores immediately after completing Cogmed® training, and participants 2 and 3 showed improved performance when tested at the third time point (three-month follow-up). For digit span backward, participant 1 showed increased scores at both the second and third time points while participants 2 and 3 exhibited improvements in scores at the second time point. For letter-number sequencing, only participant 2 showed some improvement.

**Figure 2:**
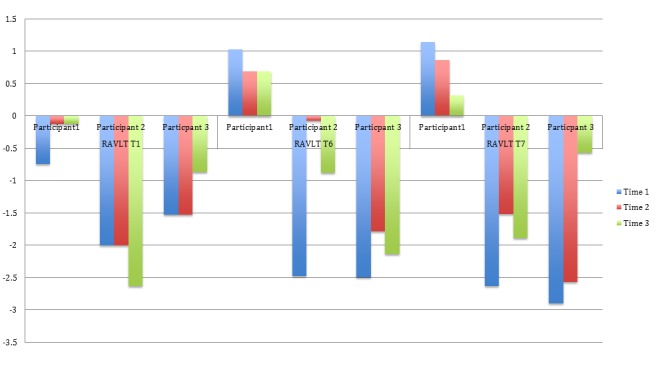
RAVLT (T1, T6, T7) Z-Scores for Three Participants Across Three Time Points

Figure 2 depicts performance on three trials of the RAVLT. Trial 1 measured attention, where participants 1 and 3 showed improvement. The SDFR trial indicates that participants 2 and 3 showed enhanced performance. The LDFR trial showed improved scores by participants 2 and 3.

**Figure 3:**
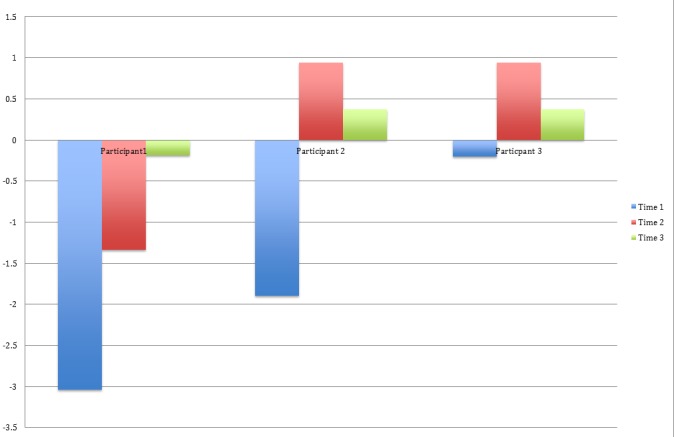
BRIEF-L Z-Scores for Three Participants Across Three Time Points

Figure 3 contains the z-scores from the Brief Test of Attention (Letters). All three participants showed an overall improvement in performance from their baseline to follow-up scores (time point 3).

**Figure 4:**
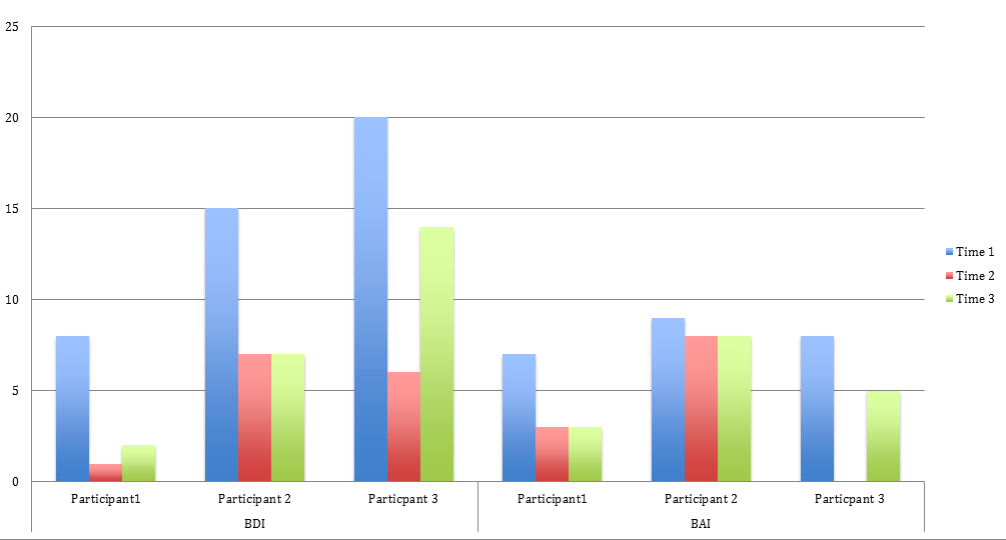
BDI and BAI Raw Scores for Three Participants Across Three Time Points

Mood was measured by the BDI and BAI. Figure 4 shows overall decreases in scores for the three participants, indicating a decrease in reported depression and anxiety.

## Conclusions

While this is preliminary data, it suggests that CRT merits further investigation for its role in facilitating post-illness functioning for survivors. The present investigators are actively recruiting patients with relatively good prognoses to study the efficacy of Cogmed® in helping restore functioning, assisting with reintegration, and consequently improving the quality of life. CRT is an essential part of the continuity of care for post-treatment neurosurgical patients in order to enhance the quality of life and assist with reintegration into vocational and/or academic environments. Computerized CRT allows access to these interventions and warrants further investigation to establish its efficacy and effectiveness.
